# An alternative interpretation of residual feed intake by phenotypic recursive relationships in dairy cattle

**DOI:** 10.3168/jdsc.2021-0080

**Published:** 2021-09-23

**Authors:** Xiao-Lin Wu, Kristen L. Parker Gaddis, Javier Burchard, H. Duane Norman, Ezequiel Nicolazzi, Erin E. Connor, John B. Cole, Joao Durr

**Affiliations:** 1 Council on Dairy Cattle Breeding, Bowie, MD 20716; 2 Department of Animal and Dairy Sciences, University of Wisconsin, Madison 53706; 3 Department of Animal and Food Sciences, University of Delaware, Newark 19716; 4 USDA, Agricultural Research Service, Animal Genomics and Improvement Laboratory, Beltsville, MD 20705-2350

## Abstract

There has been increasing interest in residual feed intake (RFI) as a measure of net feed efficiency in dairy cattle. Residual feed intake phenotypes are obtained as residuals from linear regression encompassing relevant factors (i.e., energy sinks) to account for body tissue mobilization. By rearranging the single-trait linear regression, we showed a causal RFI interpretation underlying the linear regression for RFI. It postulates recursive effects in energy allocation from energy sinks on dry matter intake, but the feedback or simultaneous effects are nonexistent. A Bayesian recursive structural equation model was proposed for directly predicting RFI and energy sinks and estimating relevant genetic parameters simultaneously. A simplified Markov chain Monte Carlo algorithm was described. The recursive model is asymptotically equivalent to one-step linear regression for RFI, yet extends the analytical capacity to multiple-trait analysis.

## Highlights

• The model postulates RFI as resulting from phenotypic recursive effects from energy sinks to DMI.
• It predicts RFI genetic values and estimates genetic parameters simultaneously.
• A simplified algorithm is proposed to sample model parameters via Marko chain Monte Carlo.
• The model extends naturally to deal with heterozygous relationships between DMI and energy sinks.
• Modeling simultaneous effects between energy sinks and DMI is possible, subject to model identifiability.

## Graphical Abstract

**Figure:**
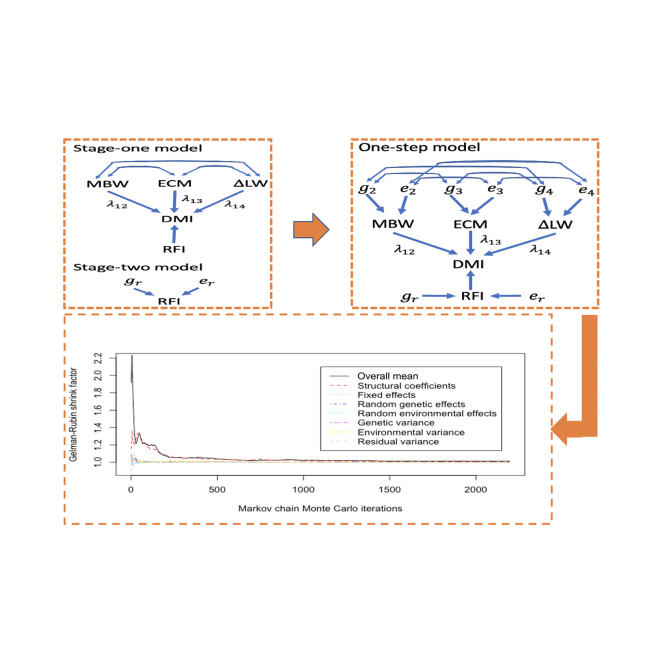
**Summary:** Genetic evaluation on residual feed intake (RFI) often takes 2 stages. Combining these 2 modeling stages leads to one-step linear regression, eliminating the need to estimate the residuals as the RFI phenotypes specifically. However, fitting phenotypes as regressor variables in a standard linear regression is criticized because phenotypes are subject to measurement errors. Multiple-trait models have been proposed, which give the genetic values of RFI through a follow-up partial regression procedure. By rearranging the linear regression equation, we came across an alternative, causal RFI interpretation by phenotype recursiveness between DMI and energy sinks. In this technical note, we propose a Bayesian recursive structural equation model (RSEM) for directly evaluating RFI, extending its analytical capacity to multiple-trait analysis.

Residual feed intake was initially proposed by Koch et al. (1963) as the residuals from linear regression (**LR**) of feed intake on various energy sinks. It represents a resource allocation theory, which partitions feed intake into the feed intake expected for the given production level and a residual portion (Herd, 2009). Genetic evaluation on RFI often takes 2 stages (Berry and Crowley, 2013). In the first stage, DMI is taken to be a linear function of variables (i.e., energy sinks) to account for body tissue mobilization. In dairy cattle, energy sinks often include metabolic body weight (**MBW**), milk net energy (**MILKNE**), and changes in BW (**ΔBW**) (e.g., Pryce et al., 2015; Tempelman et al., 2015; Løvendahl et al., 2018; Islam et al., 2020). In the second stage model, the computed RFI phenotypes are fitted by a mixed-effects model to estimate RFI genetic values and relevant genetic parameters. Combining these 2 modeling stages leads to one-step LR for RFI (e.g., Tempelman et al., 2015; Løvendahl et al., 2018), eliminating the need to estimate the residuals as RFI phenotypes specifically. Fitting phenotypes as regressor variables in LR has been criticized (Lu et al., 2015) because standard regression models assume that regressor variables have been measured precisely. In reality, however, phenotypes are subject to measurement errors. Multiple-trait models have been proposed that obtain RFI genetic values indirectly through follow-up partial regression (Kennedy et al., 1993; Lu et al., 2015; Islam et al., 2020; Tempelman and Lu, 2020). With various methods available, the biological implications for the computed RFI remain to be exploited (Martin et al., 2021).

By rearranging the LR equation, we came across an alternative, causal interpretation of RFI by phenotype recursiveness between DMI and energy sinks. Consider a single animal, say *i*. Let *y_i_*_1_ be a variable for DMI phenotypes and let *y_i_*_2_, …, *y_ik_* be variables representing the phenotypes for *k* − 1 energy sinks, all measured on this animal. A simple energy model includes only energy sinks as fixed effects plus the residual (*r_i_*) as a RFI phenotype (Løvendahl et al., 2018):

[1] $$y_{i1}=\sum_{j=2}^{k}\lambda_{1j}y_{ij}+r_{i},$$

where $\lambda_{1j}$ quantifies the effect of energy sink *j* on DMI. The energy sink model may include additional covariates or factors (Pryce et al., 2015; Tempelman et al., 2015). Then, the residual is fitted by a mixed-effects model:

[2] $${\hat{r}}_{i}=\mu_{1}+{x^{\prime }}_{i1}\beta_{1}+{z^{\prime }}_{i1}a_{1}+e_{i1},$$

where *μ*_1_ is the overall mean, **β**_1_ is a vector of fixed effects, **a**_1_ is a vector of random animal additive genetic values, **x***_i_*_1_ and **z***_i_*_1_ are the corresponding incidence vectors for animal *i*, and **e***_i_*_1_ is an error term. We did not consider random environmental effects in model [2] but they can be included similarly. Combining equations [1] and [2], and moving the energy sink phenotypes to the left-hand side of the equation, leads to

[3] $$y_{i1}-\sum_{t=2}^{k}\lambda_{1t}y_{it}=\mu_{1}+{x^{\prime }}_{i1}b_{1}+{z^{\prime }}_{i1}a_{1}+e_{i1},$$

Note that in [3], *y_i_*_1_ is a phenotype for DMI, but the fixed and random effects (i.e., **β**_1_ and **a**_1_) pertain to the system phenotype, $y_{i1}-\sum_{t=2}^{k}\lambda_{1t}y_{it},$ which is RFI. This feature contrasts that of a multiple-trait mixed effects model in which the model parameters belong to DMI and energy sinks. Hence, the recursive model can directly estimate RFI genetic values and genetic parameters without taking follow-up partial regression or reparameterization.

Next, each energy sink trait, say *j*, is described similarly by a mixed-effects model:

[4] $$y_{ij}=\mu_{j}+{x^{\prime }}_{ij}b_{j}+{z^{\prime }}_{ij}a_{j}+e_{ij},$$

By stacking equation [3] with the mixed-effects models [4] for the energy sink traits and rearranging incidence matrices and fixed and random vectors (including the residual vector), we obtain the following recursive structural equation model (**RSEM**) for individual *i*:

[5] $$\Lambda y_{i}=\mu +X_{i}\beta +Z_{i}a+e_{i},$$

where $y_{i}={\left(\begin{array}{ccc} y_{i1} & y_{i2} & \begin{array}{cc} \cdots & y_{ik} \end{array} \end{array}\right)}^{\prime },$ $\mu ={\left(\begin{array}{ccc} \begin{array}{cc} \mu_{1} & \mu_{2} \end{array} & \cdots & \mu_{k} \end{array}\right)}^{\prime },$ $e_{i}={\left(\begin{array}{ccc} \begin{array}{cc} e_{i1} & e_{i2} \end{array} & \cdots & e_{ik} \end{array}\right)}^{\prime },$ and **β** and **a** are vectors of appropriate lengths containing the fixed and random effects, respectively. The vectors of fixed and random effects are resorted for each effect by traits within individuals, and the incidence vectors are set up accordingly. Let the incidence vectors of fixed effects be the same between traits for each animal. That is, $x_{i1}=\ldots =x_{i4}=x_{i},$ where **x***_ij_* is an incidence vector linking fixed effects to the *j*th phenotype and **x***_i_* is the common incidence vector. Then, **X***_i_* = **x***_i_* ⊗ **I**, where **I** is a *k* × *k* identity matrix. Similarly, we have **Z***_i_* = **z***_i_* ⊗ **I**. Finally, the structural matrix (**Λ**) is defined as follows:

[6] $$\Lambda =\left(\begin{array}{cccc} 1 & -\lambda_{12} & \cdots & -\lambda_{1k}\\ 0 & 1 & \cdots & 0\\ \vdots & \vdots & \ddots & \vdots \\ 0 & 0 & \cdots & 1 \end{array}\right).$$

This recursive model belongs to the broad category of recursive and feedback systems for describing the phenotypic relationships between diseases and production in animal breeding (Gianola and Sorensen, 2004; Wu et al., 2007, 2008). Jamrozik et al. (2017) applied recursive modeling to analyze ratio traits (e.g., *y*_2_/*y*_1_), where *y*_1_ is taken to be the baseline trait with an assumed recursive effect on *y*_2_. Lu et al. (2015) applied a modified Cholesky decomposition of covariance matrix between DMI and 2 energy sinks. The reparameterization implied fully recursive effects from energy sinks and DMI. Neverthelesss, they did not follow structural equation modeling but retained a multiple-trait mixed-effects model and obtained partial regression coefficients from estimated covariance matrices.

The Bayesian implementation of the recursive model for RFI follows Gianola and Sorensen (2004) and Wu et al. (2007, 2010). A simplified algorithm is described below. A detailed description is available at https://redmine.uscdcb.com/documents/259). The RFI phenotypes $\left(i.e.,y_{i1}-\sum_{t=2}^{k}\lambda_{1t}y_{it}\right)$ are uncorrelated with the phenotypes of energy sinks (Kennedy et al., 1993). According to the path theory, a zero phenotypic correlation (*r_p_*) between RFI and an energy sink (indexed by *j*, for *j* = 2, …, *k*) implies either (1) $\left(1-h_{RFI}\right)\left(1-h_{j}\right)r_{e_{RFI}e_{j}}=-h_{RFI}h_{j}r_{a_{RFI}a_{j}}$ or (2) $r_{e_{RFI}e_{j}}=0$ and $r_{a_{RFI}a_{j}}=0,$ where *h* is the square root of heritability, and $r_{a_{RFI}a_{j}}$ and $r_{e_{RFI}e_{j}}$ are the genetic and residual correlations, respectively, between RFI and energy sink *j*, assuming a total determination by these 2 components. The former relationship in (1) is a strong assumption, which states that genetic and residual correlations between RFI and energy sinks were highly coordinated and they do not necessarily equal zero. We took the latter approach, option (2), by forcing the genetic (**G**) and residual (**R**) covariance between RFI and energy sinks to be zeros, because we intended to have RFI as a measure of net feed efficiency, independent of energy sinks. That is,

$$G_{0}=\left(\begin{array}{cccc} \sigma_{a_{1}}^{2} & 0 & \ldots & 0\\ 0 & \sigma_{a_{2}}^{2} & \ldots & \sigma_{a_{2}a_{k}}\\ \vdots & \vdots & \ddots & \vdots \\ 0 & \sigma_{a_{k}a_{2}} & \ldots & \sigma_{a_{k}}^{2} \end{array}\right);R_{0}=\left(\begin{array}{cccc} \sigma_{e_{1}}^{2} & 0 & \ldots & 0\\ 0 & \sigma_{e_{2}}^{2} & \ldots & \sigma_{e_{2}e_{k}}\\ \vdots & \vdots & \ddots & \vdots \\ 0 & \sigma_{e_{k}e_{2}} & .. & \sigma_{e_{k}}^{2} \end{array}\right).$$

The genetic and residual covariance matrices between DMI and energy sinks are given by

$$G_{0}^{*}=\Lambda^{-1}G_{0}\Lambda^{\prime -1};R_{0}^{*}=\Lambda^{-1}R_{0}\Lambda^{\prime -1}.$$

The conditional posterior distribution of structural coefficients does not depend on any unknown parameters of energy sinks, assuming zero genetic and residual correlations between RFI and energy sinks. This feature drastically simplifies the posterior inference of structural coefficient matrix and unknown parameters for RFI. Denote $\lambda ={\left(\lambda_{12},\lambda_{13},\cdots ,\lambda_{1k}\right)}^{\prime }.$ We assumed a multivariate normal prior distribution (MVN) for λ. That is, $\lambda |\lambda_{0},\tau^{2}\text{\textasciitilde{}}MVN\left(1\lambda_{0},I\tau^{2}\right),$ where **1** is a (*k* – 1) × 1 vector of ones, **I** is a (*k* – 1) × 1 (*k* – 1) identity matrix, and $\lambda_{0}$ and τ^2^ are hyperparameters. Then, the conditional posterior distribution of λ is also an MVN distribution (Gianola and Sorensen, 2004; Wu et al., 2007), independent of the equations for energy sinks. The conditional posterior means of λ are

[7] $$\begin{array}{l} E\left(\lambda |else\right)=\\ {\left(\begin{array}{cc} \begin{array}{cc} \begin{array}{c} \begin{array}{c} \sum_{i=1}^{n}y_{i2}^{2}+\sigma_{e_{1}}^{2}\tau^{-2}\\ \sum_{i=1}^{n}y_{i3}y_{i2} \end{array}\\ \begin{array}{c} \vdots \\ \sum_{i=1}^{n}y_{ik}y_{i2} \end{array} \end{array} & \begin{array}{c} \begin{array}{c} \sum_{i=1}^{n}y_{i2}y_{i3}\\ \sum_{i=1}^{n}y_{i3}^{2}+\sigma_{e_{1}}^{2}\tau^{-2} \end{array}\\ \begin{array}{c} \vdots \\ \sum_{i=1}^{n}y_{ik}y_{i3} \end{array} \end{array} \end{array} & \begin{array}{cc} \begin{array}{c} \begin{array}{c} \cdots \\ \cdots \end{array}\\ \begin{array}{c} \ddots \\ \cdots \end{array} \end{array} & \begin{array}{c} \begin{array}{c} \sum_{i=1}^{n}y_{i2}y_{ik}\\ \sum_{i=1}^{n}y_{i3}y_{ik} \end{array}\\ \begin{array}{c} \vdots \\ \sum_{i=1}^{n}y_{ik}^{2}+\sigma_{e_{1}}^{2}\tau^{-2} \end{array} \end{array} \end{array} \end{array}\right)}^{-1}\times \left(\begin{array}{c} \begin{array}{c} \sum_{i=1}^{n}y_{i2}w_{i1}+\sigma_{e_{1}}^{2}\tau^{-2}\lambda_{0}\\ \sum_{i=1}^{n}y_{i3}w_{i1}+\sigma_{e_{1}}^{2}\tau^{-2}\lambda_{0} \end{array}\\ \begin{array}{c} \ddots \\ \sum_{i=1}^{n}y_{ik}w_{i1}+\sigma_{e_{1}}^{2}\tau^{-2}\lambda_{0} \end{array} \end{array}\right), \end{array}$$

where *E* is the expectation, ***else*** represents the data and all other unknown model parameters, and $w_{i1}=y_{i1}-\left(\mu_{1}+{x^{\prime }}_{i1}\beta_{1}+{z^{\prime }}_{i1}a_{1}\right),$ for *i* = 1, …, *n*. Similarly, the conditional posterior distribution of location parameters (i.e., fixed and random effects) and scaling parameters (variance components), respectively, for RFI does not involve any unknown parameters for energy sinks either.

To see the link between the recursive model and linear regression for RFI, consider equation [3] and replace the structural coefficients, $\lambda_{1j},$ by partial regression coefficients, *b_j_*, for *j* = 2, …, *k*. If we move all the fixed and random effects to the left-hand side of the equation and keep the energy sinks and the residual on the right-hand side, it becomes

[8] $$y_{i1}-\mu_{1}-x\prime_{i1}\beta_{1}-{z^{\prime }}_{i1}a_{1}=\left(\begin{array}{ccc} y_{i2} & y_{i3} & \begin{array}{cc} \cdots & y_{ik} \end{array} \end{array}\right)\left(\begin{array}{c} b_{2}\\ b_{3}\\ \vdots \\ b_{k} \end{array}\right)+e_{1}.$$

Then, the least-squares solutions of the partial regression coefficients are as follows:

[9] $$\left(\begin{array}{l} {\hat{b}}_{2}\\ {\hat{b}}_{3}\\ \vdots \\ {\hat{b}}_{k} \end{array}|\mu_{1},\beta_{1},\alpha_{1}\right)={\left(\begin{array}{cccc} \sum_{i=1}^{n}y_{i2}^{2} & \sum_{i=1}^{n}y_{i2}y_{i3} & \cdots & \sum_{i=1}^{n}y_{i2}y_{ik}\\ \sum_{i=1}^{n}y_{i3}y_{i2} & \sum_{i=1}^{n}y_{i3}^{2} & \cdots & \sum_{i=1}^{n}y_{i3}y_{ik}\\ \vdots & \vdots & \ddots & \vdots \\ \sum_{i=1}^{n}y_{ik}y_{i2} & \sum_{i=1}^{n}y_{ik}y_{i3} & \cdots & \sum_{i=1}^{n}y_{ik}^{2} \end{array}\right)}^{-1}\left(\begin{array}{c} \sum_{i=1}^{n}y_{i2}w_{i1}\\ \sum_{i=1}^{n}y_{i3}w_{i1}\\ \ddots \\ \sum_{i=1}^{n}y_{ik}w_{i1} \end{array}\right),$$

where $w_{i1}=y_{i1}-\mu_{1}-{x^{\prime }}_{i1}\beta_{1}-{z^{\prime }}_{i1}a_{1}.$ Note that [7] coincides precisely with [9] if we let $\tau^{2}\to \infty$ in [7], which is equivalent to assigning flat priors to structural coefficients in [9]. In other words, the conditional posterior means of structural coefficients agree with (or are asymptotically equivalent to) the partial regression coefficients in one-step LR, given *μ*_1_, **β**_1_, and **a**_1_, if we ignore the prior values (or the impact of priors diminishes when the data dominate the posteriors). Likewise, the same conclusion holds for the location and scaling parameters between the recursive model and one-step linear regression for RFI.

The data set consisted of 645 first-parity cows with phenotypes, derived from 125 sires and 477 dams, and raised in the USDA Beltsville Agricultural Research Center (BARC) Dairy Herd (Beltsville, MD). The phenotypic data included DMI, MBW, MILKNE, and ΔBW, all obtained as averages over a 42-d trial. Their means (SD) were 28.9 (3.81) kg/d, 113.8 (6.71) kg^0.75^, 21.1 (2.18) Mcal, and 0.47 (0.22) kg. Phenotypes were standardized to means of zero and unit variance to facilitate comparing the estimated effects between traits not affected by the units of the traits. The data standardization did not change the phenotypic correlations, which were 0.441 (DMI vs. MBW), 0.556 (DMI vs. MILKNE), 0.166 (DMI vs. ΔBW), 0.132 (MBW vs. MILKNE), 0.193 (MBW vs. ΔBW), and −0.036 (MILKNE vs. ΔBW). We compared 2-stage models and one-step models for RFI, implemented by LR and Bayesian RSEM, respectively. Model LR1 was the stage-one model of the 2-stage linear regression, with MBW, MILKNE, and ΔBW as fixed effects. Model LR2 was a one-step linear regression with 3 energy sinks and DIM (DIM = 71, 72, 73, 74, 75, 76, 77) as the fixed effects and individual animal effects as random variables. Model LR3 had all the model parameters in LR2, plus test weeks (i.e., 143 levels) as an nongenetic random variable. Models RSEM1, RSEM2, and RSEM3 were the Bayesian recursive equation models of LR1, LR2, and LR3, respectively, but with phenotypic recursive effects assumed from energy sinks to DMI. For a Bayesian recursive model, we ran 30 parallel Markov chain Monte Carlo (MCMC) chains, each consisting of 2,200 iterations, with a burn-in of 2,000 iterations and thinned every 2 iterations. We also ran single-trait mixed-effects model analyses on each of these traits, and a multiple-trait, mixed-effects model (**MT**), with DIM as a fixed effect and test-week and animal effects as random effects. Markov chain Monte Carlo convergence was examined for the model parameters using the shrink factor (Gelman and Rubin, 1992). The MCMC chains, which were initialized randomly, converged quickly. The shrink factor dropped below 1.1 after 200 iterations and approached 1.0 after 1,000 iterations (see the graphical abstract). Saved posterior samples after 1,000 iterations were pooled and used to make the posterior inference of unknown parameters.

The estimated effects from energy sinks to DMI (Table 1) agreed well between LR and recursive models with similar settings (e.g., between LR1 and RSEM1). On standardized phenotypic scales, MILKNE had the largest effects on DMI (0.51 to 0.53), followed by MBW (0.31 to 0.35), and ΔBW had the smallest effect on DMI (0.12 to 0.13). Including different sets of fixed and random effects led to varied RFI definitions, and the estimated partial regression coefficients (or structural coefficients) varied accordingly. Nevertheless, the estimated RFI genetic values agreed very well between a 2-stage model and a one-step model. The Spearman correlation of the estimated RFI genetic values was close to 1 between LR1 and LR3 and between RSEM1 and RSEM3, and rerankings happened rarely. The Spearman correlation between LR3 and RSEM3 was 0.998 (Figure 1, panel A). The differences were primarily due to Monte Carlo errors.

**Table 1:** Mean or posterior mean (SD or posterior SD) of the estimated effects of 3 energy sinks on DMI, obtained using different models

Model	Effects from energy sinks to DMI^1^		
	MBW	MILKNE	ΔBW
LR^2^			
LR1	0.351 (0.029)	0.514 (0.029)	0.117 (0.030)
LR2	0.331 (0.030)	0.523 (0.029)	0.123 (0.029)
LR3	0.312 (0.029)	0.534 (0.028)	0.126 (0.030)
RSEM^3^			
RSEM1	0.351 (0.030)	0.514 (0.029)	0.117 (0.030)
RSEM2	0.331 (0.030)	0.522 (0.029)	0.124 (0.029)
RSEM3	0.311 (0.030)	0.530 (0.029)	0.126 (0.030)
MT^4^			
Phenotypic	0.351	0.514	0.117
Genetic	0.257	0.746	1.185

1 MBW = metabolic BW; MILKNE = milk net energy; ΔBW = change in BW.

2 LR1 = linear regression with 3 energy sinks (MBW, MILKNE, and ΔBW) as the fixed effects; LR2 = linear regression with DIM and 3 energy sinks as fixed effects and individual animal genetic values as random effects; LR3 = LR2 plus test weeks (TW) as a nongenetic random variable.

3 RSEM1–3 = Bayesian recursive models having the same set of model effects as LR1–3.

4 Phenotypic/Genetic = partial regression coefficients based on phenotypic and genetic variance-covariance matrices from a multiple-trait model (MT), respectively. DIM were included as fixed effects and TW and individual animal genetic values were included as random effects.

**Figure 1:**
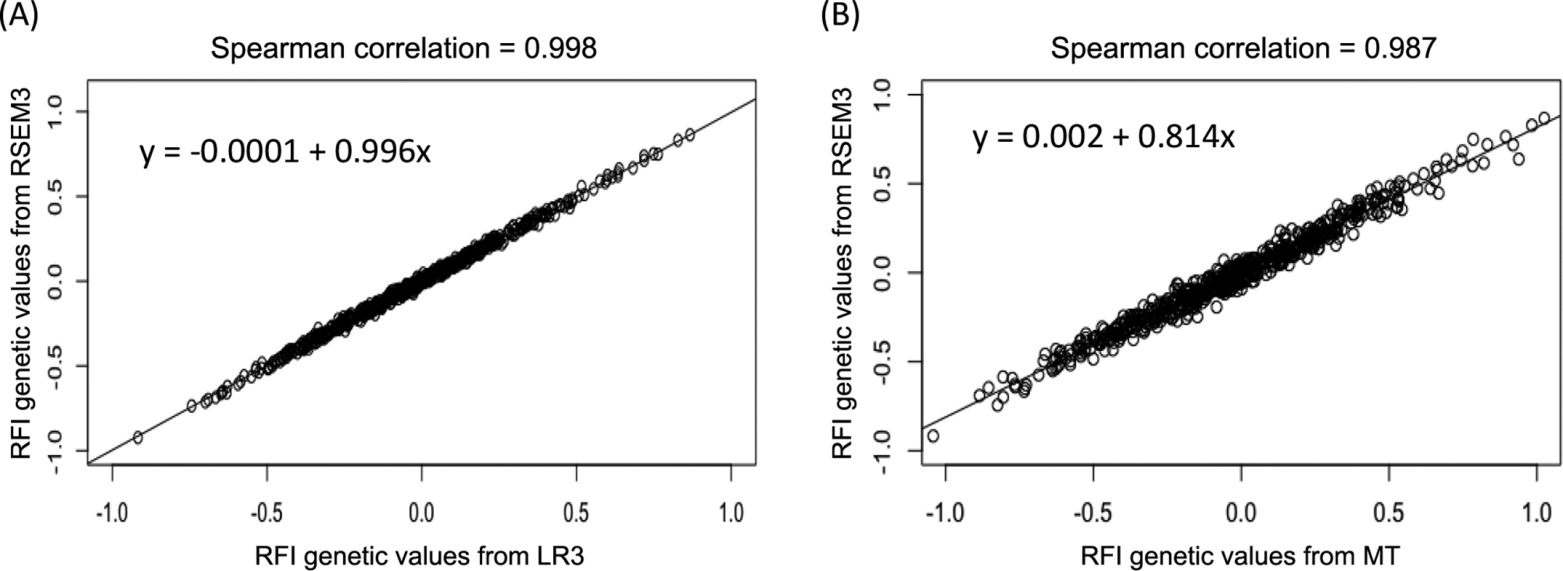
Spearman correlation plots of the estimated genetic animal values obtained from different models: (A) Recursive structural equation model (RSEM3) versus one-step linear regression (LR3); (B) RSEM3 versus multiple-trait mixed-effects model (MT).

The MT model allows for distinguishing between genetic and residual effects. The genetic partial regression coefficients did not agree with the partial regression coefficients (or structural coefficients) obtained from single-trait LR (or recursive models). Nevertheless, the partial regression coefficients estimated from phenotypic (co)variance agreed very well with the partial regression coefficients from LR1 (or the structural coefficients from RSEM1). The multiple-trait, mixed-effects model assumed correlational relationships between the traits, which has no causal interpretation. Nevertheless, a fully-recursiveness system can be assumed based on the modified Cholesky decomposition (Lu et al., 2015). Consider the phenotypic relationships, for example, in the present example. The $L\Sigma L^{\prime }$ decomposition implies fully recursive relationships for the traits (ordered by *y_i_*_4_, *y_i_*_3_, *y_i_*_2_, and *y_i_*_1_). Here, **L** is the unit lower triangular matrix, which corresponds to the structural coefficient matrix in RSEM, as follows:

[10] $$\Lambda^{\prime }=L^{\prime }=\left(\begin{array}{cc} \begin{array}{cc} \begin{array}{c} \begin{array}{c} 1\\ 0 \end{array}\\ \begin{array}{c} 0\\ 0 \end{array} \end{array} & \begin{array}{c} \begin{array}{c} -b_{12}\\ 1 \end{array}\\ \begin{array}{c} 0\\ 0 \end{array} \end{array} \end{array} & \begin{array}{cc} \begin{array}{c} \begin{array}{c} -b_{13}\\ -b_{23} \end{array}\\ \begin{array}{c} 1\\ 0 \end{array} \end{array} & \begin{array}{c} \begin{array}{c} -b_{14}\\ -b_{24} \end{array}\\ \begin{array}{c} -b_{34}\\ 1 \end{array} \end{array} \end{array} \end{array}\right),$$

where $b_{jj^{\prime }}$ is the effect (i.e., partial regression coefficient) from trait *j*′ to trait *j*, and $y_{ij}=\sum_{j^{\prime }=1}^{j-1}b_{jj^{\prime }}y_{ij^{\prime }},$ for *j* = 2, …, 4. The covariance matrix between the reparameterized variables (*y_i_*_4_, *y_i_*_3_ – *b*_34_*y_i_*_4_, $y_{i2}-\sum_{j=3}^{4}b_{1j}y_{ij},$ and $y_{i1}-\sum_{j=2}^{4}b_{1j}y_{ij}$) is diagonal, meaning they are mutually independent. Following the same Bayesian modeling settings to implement the reparameterized MT model as a Bayesian recursive model, we can show that *b*_12_, *b*_13_, and *b*_14_ are estimated identical to those for $\lambda_{12},$ $\lambda_{13},$ and $\lambda_{14}$ in [7]. However, they differed in the assumed relationships between energy sinks. For example, the model by Lu et al. (2015) assumed recursive effects from milk energy on MBW, and the relationships between energy sinks are correlational in a recursive model. The estimated partial regression coefficients from the MT model based on the phenotypic covariance matrix coincided precisely with the structural coefficients, showing differences only after the third decimal point, but they can vary depending on the data.

Overall, the heritability estimates obtained from RSEM3 and single-trait LR were moderate to high for DMI (0.40–0.49) and MBW (0.59) but low for ΔBW (0.002–0.04; Table 2). The heritability estimate for RFI was 0.392 by one-step LR and 0.240 by RSEM3. The heritability estimates for energy sinks were within comparable ranges of previous studies (e.g., Berry and Crowley, 2013; Tempelman et al., 2015). The RFI heritability estimates were similar to those reported by Connor et al. (2013). They reported an RFI heritability of 0.36 using only the USDA AGIL (Animal Genomics and Improvement Laboratory) data for early lactation cows. Tempelman et al. (2015) reported lower RFI heritability estimates (0.18 ± 0.02) and country-specific estimates ranging from 0.06 to 0.24. Residual feed intake heritability was 0.16 in data sets that included the AGIL data (Lu et al., 2015; Li et al., 2020). Genetic correlations were moderate to high between DMI and RFI, MBW, and MILKNE (0.44 to 0.72), and low between DMI and ΔBW (0.13–0.20) and between MBW and MILKNE (0.15–0.16) (Table 2). The genetic correlations between RFI and energy sinks were forced to be zeros with RSEM3, but they had small values (−0.03 to 0.01) based on the multiple-trait model.

**Table 2:** Heritability estimates and genetic correlations for DMI, energy sinks, and residual feed intake, obtained using different models^1^

Item	DMI	MBW	MILKNE	ΔBW	RFI
Heritability					
ST-LR^2^	0.489	0.592	0.355	0.044	0.392
RSEM3^3^	0.400 (0.028)	0.589 (0.021)	0.190 (0.025)	0.002 (0.027)	0.240 (0.038)
Genetic correlations^4^					
DMI	1	0.434 (0.060)	0.604 (0.055)	0.129 (0.090)	0.717
MBW	0.454	1	0.145 (0.126)	0.184 (0.150)	0
MILKNE	0.576	0.161	1	−0.089 (0.133)	0
ΔBW	0.119	0.206	−0.086	1	0
RFI	0.713	−0.022	0.012	−0.033	1

1 MBW = metabolic BW; MILKNE = milk net energy; ΔBW = change in BW; RFI = residual feed intake.

2 ST-LR (MT) = single trait (multiple-trait) mixed-effects model with DIM as the fixed effects, plus test weeks and individual animal genetic values as the random effects; RSEM3 = Bayesian recursive model having DIM and 3 energy sinks (MBW, MILKNE, and ΔBW) as the fixed effects, plus test weeks and individual animal genetic values as the random effects.

3 Posterior standard deviations are shown in parentheses.

4 Genetic correlations obtained from RSEM3 (above diagonal) and MT (below diagonal).

The present study assumed a single, homogeneous structural coefficient matrix. Model expansion to account for heterogeneous structural coefficient matrices is straightforward, where the conditional distributions for structural coefficients take the same formula but are sampled separately for each subpopulation (Wu et al., 2010). The MT model also allows for distringuishing between genetic and residual relationships. Which assumption is more plausible remains a topic for further investigation. Modeling simultaneous effects between energy sinks and DMI and between energy sinks is possible, subject to the model identifiability.
